# The MAPKKKs Ste11 and Bck1 jointly transduce the high oxidative stress signal through the cell wall integrity MAP kinase pathway

**DOI:** 10.15698/mic2015.09.226

**Published:** 2015-09-07

**Authors:** Chunyan Jin, Stephen K. Kim, Stephen D. Willis, Katrina F. Cooper

**Affiliations:** 1 Department of Molecular Biology, Rowan University School of Osteopathic Medicine, Stratford, NJ, 08055 USA.

**Keywords:** cyclin C, oxidative stress, protein degradation, programmed cell death, MAPK signal transduction pathways, mitochondrial morphology

## Abstract

Oxidative stress stimulates the Rho1 GTPase, which in turn induces the cell wall integrity (CWI) MAP kinase cascade. CWI activation promotes stress-responsive gene expression through activation of transcription factors (Rlm1, SBF) and nuclear release and subsequent destruction of the repressor cyclin C. This study reports that, in response to high hydrogen peroxide exposure, or in the presence of constitutively active Rho1, cyclin C still translocates to the cytoplasm and is degraded in cells lacking Bck1, the MAPKKK of the CWI pathway. However, in mutants defective for both Bck1 and Ste11, the MAPKKK from the high osmolarity, pseudohyphal and mating MAPK pathways, cyclin C nuclear to cytoplasmic relocalization and destruction is prevented. Further analysis revealed that cyclin C goes from a diffuse nuclear signal to a terminal nucleolar localization in this double mutant. Live cell imaging confirmed that cyclin C transiently passes through the nucleolus prior to cytoplasmic entry in wild-type cells. Taken together with previous studies, these results indicate that under low levels of oxidative stress, Bck1 activation is sufficient to induce cyclin C translocation and degradation. However, higher stress conditions also stimulate Ste11, which reinforces the stress signal to cyclin C and other transcription factors. This model would provide a mechanism by which different stress levels can be sensed and interpreted by the cell.

## INTRODUCTION

A conserved survival instinct in all living cells is the ability to adapt to changes in their extracellular environment. To achieve this, cells convey information from a sensory input via a signaling cascade to the genes in charge of orchestrating the cellular response. Among the signaling pathways, the mitogen activated kinase cascades (MAPKs) are very well conserved from yeast to mammals (reviewed in 1). Each MAPK cascade consists of a canonical module of three protein kinases, a MAP kinase kinase kinase (MAPKKK/MEKK), a MAP kinase kinase (MAPKK/MEK) and a MAP kinase (MAPK). In *S. cerevisiae*, there are five known MAPK cascades (reviewed in 2,3) that control responses to different external signals stresses including nutrient starvation, high osmolarity, mating pheromone, and oxidative stress. Traditionally, the MAPK pathways have been depicted as insulated with each cascade being activated only in response to a particular extracellular signal. For example, the high osmolarity glycerol (HOG) pathway is predominantly (but not exclusively) activated in response to changes in osmolarity (reviewed in 4), whereas the cell wall integrity (CWI) pathway is triggered by numerous stresses including cell wall deterioration, temperature shifts and oxidative stress (reviewed in 5,6). More recently, it has been suggested that in situations where signal strength exceeds pathway capacity, there is a crosstalk between the pathways 7,8.

**Figure 1:**
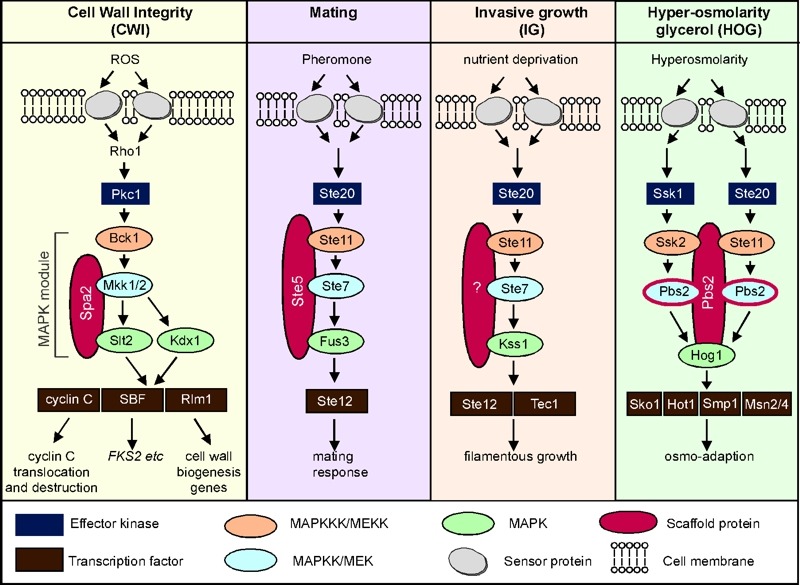
FIGURE 1: Schematic diagram of the MAPK modules of the cell wall integrity (CWI), mating, invasive growth (IG) and hyper-osmolarity glycerol (HOG) pathways in *S. cerevisiae*.

Ste11 is a shared MAPKKK that plays an important role in transducing several signals. Ste11 transduces signals important for mating, invasive growth (IG, also known as pseudohyphal pathway) and resistance to high osmolarity, even though each pathway responds to different external stimuli and produces different outputs (see Figure 1). Ste11 is activated by the yeast homolog of the p21-like kinase (PAK) called Ste20 9. Dependent on the stimuli received, Ste11 phosphorylates a different MAPKK. How the cell determines which MAPKK to activate is only partially understood but thought to be dependent upon upstream components which route the signal to the appropriate MAPKK 3,10. Over the last two decades, a principle has emerged involving scaffold proteins, which physically assemble MAPK modules, in directing which MAPK module is activated following a particular stress (reviewed in 11). However, the Ste5 scaffold mediating the mating pathway (see Figure 1) was shown to function as a conformational switch to gate the flow of information between the mating and IG pathways 12. These results indicate that the role scaffolds play in sorting MAPK signaling may be more complicated.

In addition to signaling in the HOG, IG and mating pathways, Ste11 is also required for the expression of *FKS2* 13,14,15*. FSK2 *encodes one of two alternate stress-induced catalytic subunits of the beta-1,3 glucan synthase complex that is important for cell wall integrity under conditions of cell wall stress 16. The role Ste11 plays in its activation is complex but it has been shown that its activation can be mediated by Ste11 cross talking with the mating, HOG, calcineurin and CWI pathways 13,15. Thus, although the molecular mechanism by which this cross-talk is achieved is not well defined, it seems clear that Ste11 has functional redundancy with other MAPKK's 14,15 (see Figure 1).

The CWI pathway is normally triggered by factors that evoke cell wall damage including oxidative stress (reviewed in 6). The cell wall sensors signal through Rho1 to protein kinase C 17,18 that in turn stimulates the CWI MAPK module. In brief, the stress signal flows from Pkc1 to Bck1 (MEKK), the redundant MEKs Mkk1 or Mkk2 (written hereafter Mkk1/2) to finally the Slt2/Mpk1 MAP kinase (see 6 for review). One readout of the CWI pathway is the activation of two transcription factors, Rlm1 and SBF, which induce the expression of genes that remodel the cell wall 19,20,21,22. Rlm1 is activated by Slt2 mediated phosphorylation 20 whereas Slt2, and its pseudokinase paralogue Kdx1, use a noncatalytic mechanism to activate SBF 23,24,25.

We have identified a third transcription factor called cyclin C that is regulated by the CWI pathway (26,27,28 and reviewed in 29). Unlike cyclins that regulate cell cycle progression, cyclin C and its cyclin dependent partner Cdk8, are conserved members of the Mediator complex associating with the RNA polymerase II holoenzyme (reviewed in 30). Cyclin C-Cdk8 predominantly acts as a transcriptional repressor, regulating over 100 loci including many stress response genes 31,32. To relieve this repression, cyclin C, but not Cdk8, is translocated to the cytoplasm where it is consequently degraded in an ubiquitin dependent manner 33,34. Importantly, before it is destroyed, cyclin C is required for stress-induced mitochondrial fission and programmed cell death 34,35. This cytoplasmic role of cyclin C in mediating mitochondrial fission and intrinsic apoptosis is conserved in mammals 36.

In this report, we show that under conditions of high oxidative stress, the capacity of the canonical CWI MAPK module is exceeded. In short, we provide evidence that Slt2 can be activated following high oxidative stress in the absence of Bck1. As a result, cyclin C is both translocated to the cytoplasm and degraded. This Bck1 independent translocation of cyclin C requires Ste11. Furthermore, we provide evidence that the normal stress-induced path for cyclin C translocation starts in the nucleus, then transits to the nucleolus and finally enters the cytoplasm. We also show that CWI MAPK activity is only required for nucleolus-cytoplasm translocation and not for the nuclear to nucleolus movement. These results suggest a mechanism by which high-stress conditions are recognized differently by the cell and transduced into changes in both gene expression and mitochondrial dynamics.

## RESULTS

### Bck1 is not required for MAPK mediated degradation of cyclin C under high stress

We have previously shown that the conserved CWI MAPK pathway transduces the oxidative stress signal from the cell wall sensors to induce cyclin C nuclear to cytoplasmic translocation and destruction 27. In that report, a model was proposed in which the strength of the stress dictates which sensors are required. Here we examined whether the severity of the stress damage dictates the route the signal takes to trigger cyclin C relocalization.

**Figure 2:**
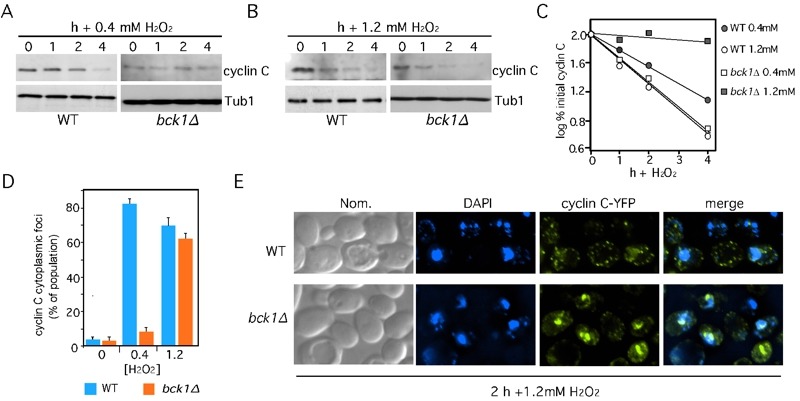
FIGURE 2: Bck1 is not required for cyclin C nuclear to cytoplasmic translocation and degradation following high (1.2 mM) H_2_O_2_ stress. Wild type (RSY10) and *bck1*∆ (RSY1050) cultures expressing myc-cyclin C (pLR337) were grown to mid-log phase (0 h) then treated with 0.4 mM **(A)** or 1.2 mM **(B)** H_2_O_2_ for the indicated times. Cyclin C levels were determined by Western blot analysis of immunoprecipitates. Tub1 levels were used as a loading control. **(C)** Quantification of the results obtained in (A) and (B). **(D)** The percent of cells (mean ± s.e.m.) within the population displaying at least 3 cytoplasmic cyclin C foci is given before and following H_2_O_2_ (0.4 and 1.2 mM) treatment for 2 h. At least 200 cells were counted per time point from 3 individual isolates. **(E)** Representative images (collapsed de-convolved 2 µm slices) of WT and *bck1*∆ cells harboring cyclin C-YFP and after 2 h 1.2 mM H_2_O_2_ stress.

To test this, we first examined whether the MEK kinase in the CWI pathway, Bck1, is required to transduce low and high oxidative stress signal to cyclin C. To address this question, we used functional cyclin C-myc and cyclin C-YFP reporters to examine cyclin C degradation kinetics and cytoplasmic localization, respectively. Consistent with our previously published work 26,28,34, Bck1 is required for cyclin C destruction and cytoplasmic relocalization of cyclin C following low oxidative stress (0.4 mM H_2_O_2_) exposure (Figure 2A, 2C and 2D). However, cyclin C destruction occurs with kinetics similar to wild type in a *bck1*∆ mutant exposed to 1.2 mM H_2_O_2_ (Figure 2B, quantitated in Figure 2C). As anticipated from these results, we find that cyclin C-YFP forms cytoplasmic foci in the *bck1*∆ mutant exposed to 1.2 mM H_2_O_2_ (Figure 2D, see Figure 2E for representative images). We also observed that cyclin C localization in 80% of *bck1*∆ cells is both just outside of the nucleus in a perinuclear location as well as in the cytoplasm. This intermediate and cyclin C localization phenotype (see images in Figure S1A) is however also observed in 28% of wild type cells after 2 h stress and this number decreases to 5% after 3 h H_2_O_2_ stress. This suggests that the “intermediate phenotype” is a transient state in the kinetics of cyclin C export. Taken together these results indicate that an additional signaling pathway(s) is employed to transmit the oxidative stress signal when cells are exposed to elevated H_2_O_2_ concentrations.

### Cyclin C destruction following high levels of oxidative stress requires both MAPKs of the CWI pathway

**Figure 3:**
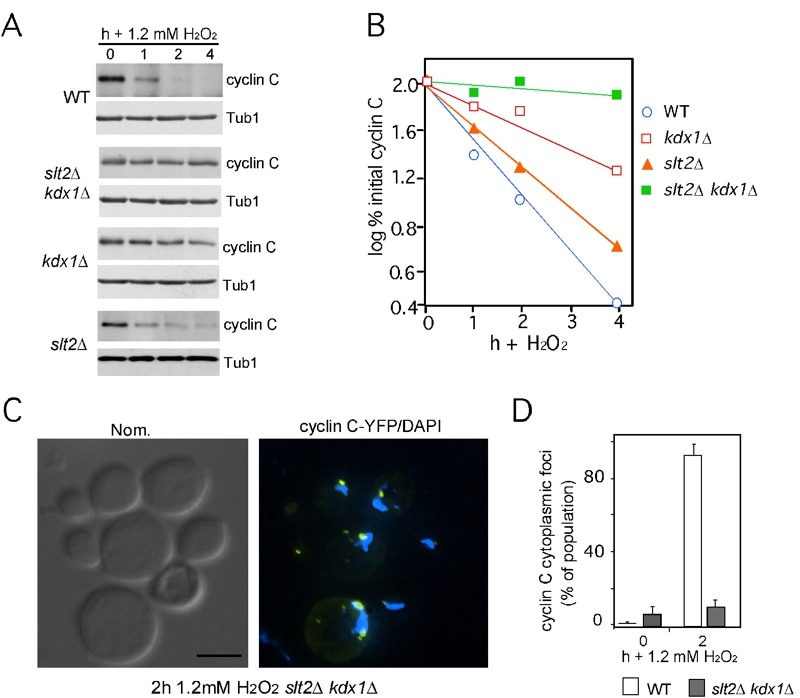
FIGURE 3: Kdx1 and Slt2 promote cyclin C relocalization and destruction in response to 1.2 mM H_2_O_2_ stress. **(A)** Cyclin C levels were monitored as described previously in wild type (RSY10), *slt2*∆* kdx1*∆ (RSY1737),* slt2*∆ (RSY1006) and* kdx1*∆ (RSY1736) strains exposed to 1.2 mM H_2_O_2_ for the times indicated. Tub1 levels were used as a loading control. **(B)** Quantification of the results obtained in (A). **(C)** Cyclin C remains perinuclear in the *slt2*∆* kdx1*∆ strain following 1.2 mM H_2_O_2_ stress. Fluorescence microscopy was conducted on mid-log phase on *slt2*∆* kdx1*∆ cells expressing YFP-cyclin C (pBK37) following (2 h) 1.2 mM H_2_O_2_ treatment. The cells were fixed, stained with DAPI and then examined by fluorescence microscopy. Representative images (collapsed deconvolved 0.2 µm slices) of the results obtained are shown. **(D)** Quantification of the results in C. The percent of the population displaying at least 3 cyclin C-YFP foci in the cytoplasm is given (mean ± s.e.m.). At least 200 cells were counted per time point from 3 individual isolates.

Both Slt2 and its pseudokinase Kdx1 mediate the export and degradation of cyclin C under conditions of low H_2_O_2_ stress 28. The results described above indicate that a Bck1 independent pathway also transmits the high-stress signal to cyclin C. To determine whether downstream components of the CWI pathway are required for transducing the high-stress signal, cyclin C levels were monitored in a *slt2*∆* kdx1*∆ strain. These results show that cyclin C is stable following 1.2 mM H_2_O_2_ exposure whereas it is degraded in the single mutants albeit with slower kinetics (Figure 3A, quantitated in Figure 3B). As cyclin C is normally destroyed following its translocation to the cytoplasm, we monitored cyclin C-YFP localization in the *slt2*∆* kdx1*∆ strain subjected to 1.2 mM H_2_O_2_ treatment. Interestingly, fluorescence microscopy revealed that cyclin C-YFP did not form multiple cytoplasmic foci (Figure 3C and S1A). Rather, a single peri-nuclear focus was observed in most cells (quantitated in Figure 3D and see Figure S1B for field view). However, cyclin C was exported in *slt2*∆ cells after treatment with 1.2 mM H_2_O_2_ (Figure S1C and D). These results indicate that cyclin C is able to translocate from diffuse nuclear localization to a single peri-nuclear focus but was unable to enter the cytoplasm.

### Cyclin C transits through nucleolus before it is exported into the cytoplasm

**Figure 4:**
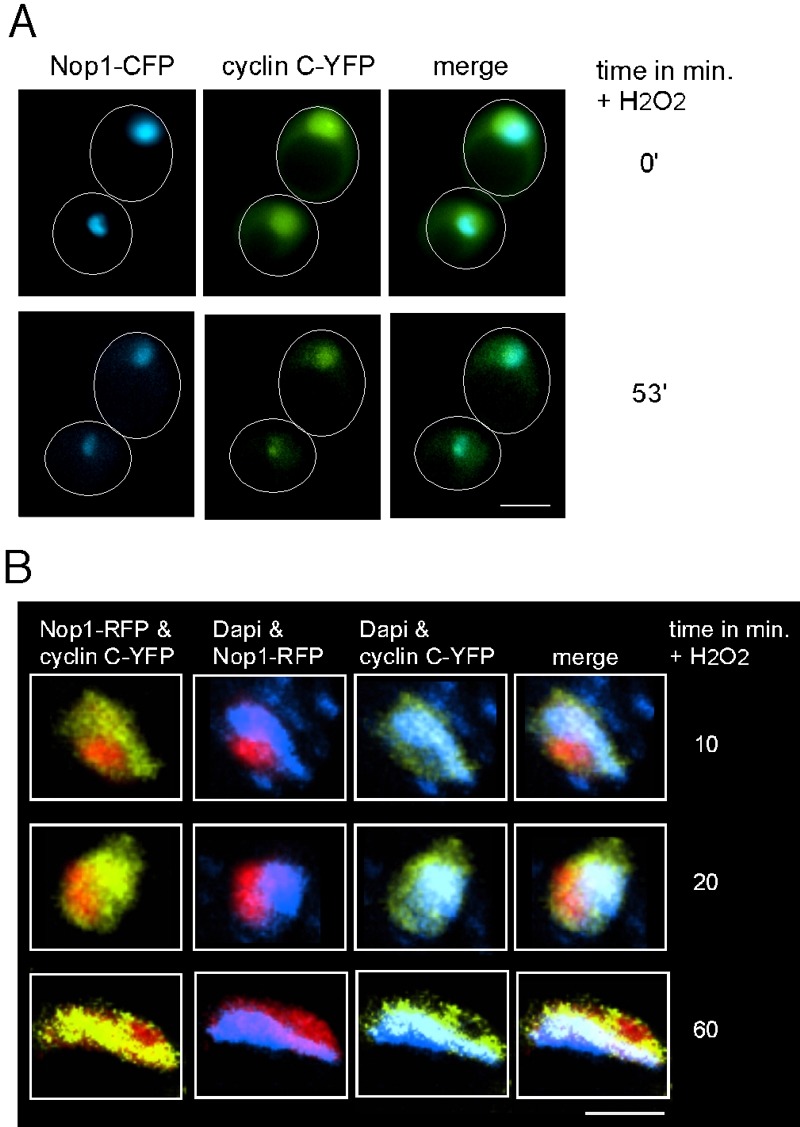
FIGURE 4: Cyclin C transits through the nucleolus in response to stress. **(A)** Live cell imaging of immobilized wild type cells (FLY1589) expressing cyclin C-YFP and Nop1-CFP before (0 min) and following (53 min) exposure to 1.0 mM H_2_O_2_. Arrows indicate restricted cyclin C-YFP signal coinciding with Nop1-CFP. **(B)** Enlarged fixed cell images of nucleus and surrounding area of wild type cells (RSY10) expressing cyclin C-YFP and Nop1-RFP. Cells were harvested and stained with DAPI following 1.2 mM H_2_O_2_ at the time points indicated.

Our previous studies revealed that in oxidatively stressed *cdk8*∆ mutants, cyclin C-YFP formed a lone peri-nuclear focus that associated with the nucleolus 35. In addition, Cdk8 also re-localized to the nucleolus in wild-type stressed cells but did not enter the cytoplasm 34. These results led to the speculation that the normal stress-induced path for cyclin C starts in the nucleoplasm, then transits to the nucleolus and finally enters the cytoplasm. However, localization to the nucleolus was only observed in mutant cells raising the possibility that the nucleolar endpoint for cyclin C may not be the normal route in wild-type cells. To test this possibility, a wild-type culture expressing the nucleolar marker Nop1-CFP and cyclin C-YFP was grown to mid-log phase, harvested, then embedded in low temperature melting agarose containing 1.0 mM H_2_O_2_ (see Materials and methods for details). Images were collected immediately after the cells were immobilized in the agarose and every minute starting at 30 min for an additional 60 min. As expected in unstressed cells, cyclin C-YFP displayed diffuse nuclear staining that may partially overlap with the Nop1-CFP signal (0 min time point, Figure 4A). However, at minute 53, the cyclin C-YFP signal became more condensed and co-localized extensively with the nucleolar marker (bottom panels, Figure 4A). However, cyclin C-YFP co-localization with the nucleolus was transient, remaining nucleolar approximately seven minutes in this experiment before entry into the cytoplasm.

These results indicate that, in response to oxidative stress, cyclin C-Cdk8p normally transit to the nucleolus with only cyclin C continuing on to the cytoplasm. To further test this model, fixed wild-type cells harboring cyclin C-YFP and Nop1-RFP were examined 10, 20 and 60 minutes after treatment with 1.2 mM H_2_O_2_. After 10 minutes, a portion of cyclin C can be seen in the nucleolus (Figure 4B, see Figure S2 for complete image of cell). Cyclin C-YFP and Nop1-RFP co-localization becomes more pronounced with time and after 60 minutes of stress most of cyclin C has moved to the nucleolus. Taken together these results suggest a model in which cyclin C first transitions to the nucleolus before it is exported to the cytoplasm. However, the transient nature of cyclin C-nucleolar co-localization makes it difficult to capture this interaction for more detailed study.

### Activated Rho1 signals cyclin C destruction through both CWI-dependent and independent pathways. 

**Figure 5:**
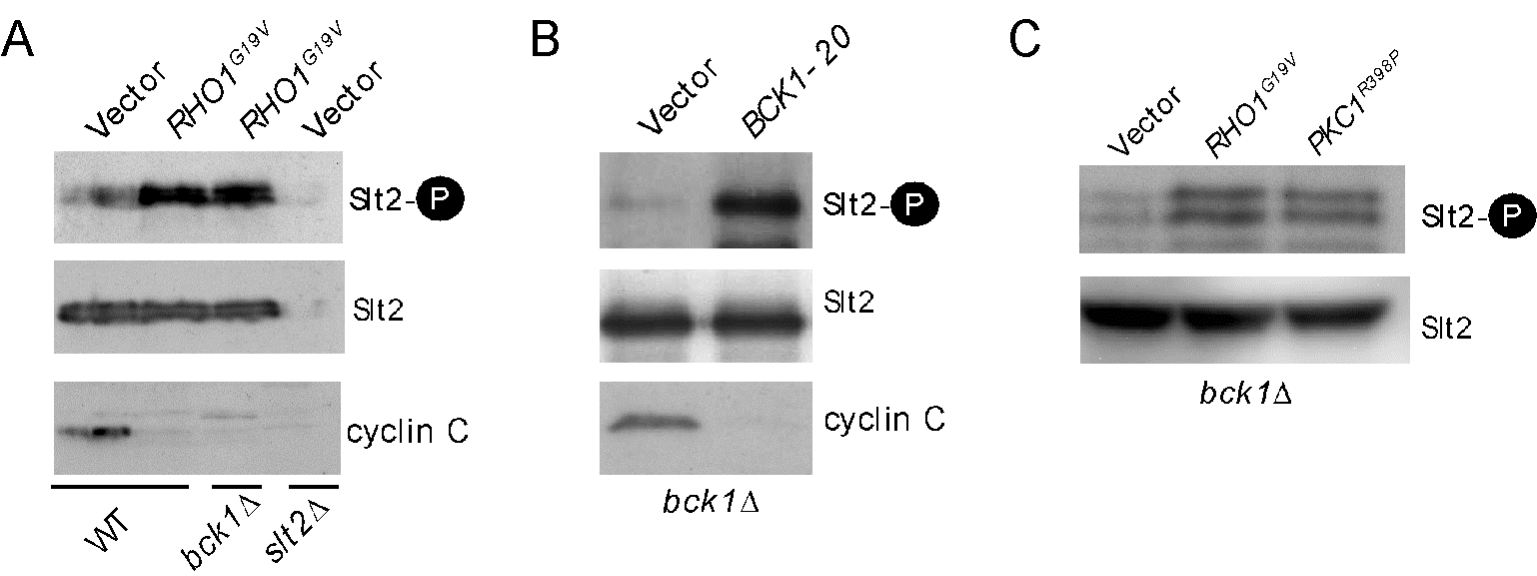
FIGURE 5: Bck1 is not required for Slt2 T-loop phosphorylation and cyclin C destruction following hyperactivation of the CWI pathway. **(A)** Slt2 T-loop phosphorylation (upper panel) was determined by Western blot analysis of protein extracts prepared from mid-log phase cultures (WT, RSY10, *bck1*∆, RSY1050, and *slt2*∆, RSY1057) expressing functional cyclin C-YFP (pBK37) and either *RHO1^G19V^* or a vector control. The blot was stripped and re-probed for Slt2 and cyclin C protein levels (middle and lower panels respectively). **(B)** As in (A) except that Slt2 and cyclin C were monitored in *bck1∆* cells expressing either a vector or a plasmid harboring a constitutively active *BCK1* allele (*BCK1-20*). **(C)** As in (A) except that Slt2 phosphorylation (top panel) was also monitored in *bck1*∆ cells expressing a hyper-active allele of *PKC1* (*PKC1^R398P^*).

Our results indicate that exposure to high H_2_O_2 _concentrations leads to cyclin C destruction through both CWI-dependent and independent pathways. These results may indicate the existence of another pathway separate from the CWI system. Conversely, the CWI pathway itself may be modified in response to high stress conditions. To test these two models, we took advantage of previous studies demonstrating that cell wall stress induces sensor clustering 37 and enhanced Rho1 activation 38. To genetically mimic this process, a constitutively activated *RHO1* allele, *RHO1^G19V^*^39^, was utilized. Protein extracts were prepared from log-phase cultures and T-loop phosphorylation was used to monitor Slt2 activation. As a downstream readout for Slt2 activation, cyclin C levels were also monitored. The results revealed that Bck1 is not required for Slt2 activation or cyclin C degradation in the presence of Rho1^G19V^ (Figure 5A). The *bck1*∆ mutant was acting as expected as introduction of a hyperactive *BCK1 *allele (*BCK1-20* 40) could efficiently induce Slt2 phosphorylation and trigger cyclin C degradation (Figure 5B). Taken together, these results indicate that hyperactivated Rho1, either by mutation, or by enhanced sensor clustering through elevated H_2_O_2_ exposure, can transmit the stress signal to cyclin C through Slt2 in a Bck1-independent manner.

GTP-bound Rho1 has six known effectors in yeast (see 41 for review) including Pkc1 (see Figure 1). Pkc1 also transmits the oxidative stress signal to Slt2 42. As *PKC1* is an essential gene 43, we monitored Slt2 T-loop phosphorylation in *bck1*∆ cells expressing a constitutively active form of Pkc1 (PKC1^R398P^ 43). The results show that Slt2 is activated under these conditions (Figure 5C). In addition, these findings suggest that Pkc1 is the effector molecule that mediates cyclin C degradation under conditions of high stress.

### Activated Rho1p induces mitochondrial fission and promotes PCD through both cyclin C-dependent and independent pathways.

**Figure 6:**
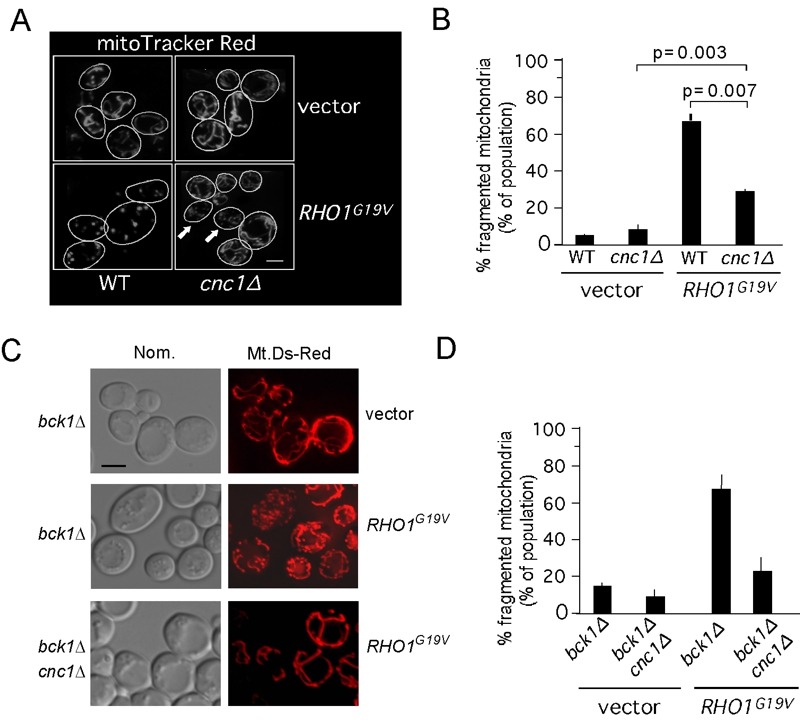
FIGURE 6: *RHO1^G19V^* promotes cyclin C-dependent mitochondrial fission and programmed cell death in the absence of stress. **(A)** Mitochondrial morphology was followed by fluorescence microscopy in wild type (RSY10) and *cnc1*∆ (RSY391) cultures expressing mt-dsRed and either a vector control or *RHO1^G19V^*. Representative images (0.2 µm slices) of each cell type are shown. The arrows indicate cells exhibiting fission in the *cnc1*∆ mutant. **(B)** Quantitation of three independent isolates exhibiting reticular or fragmented mitochondria were determined from ≥ 600 cells per culture (mean ± s.e.m.) p values for then indicated data sets are shown. **(C)** Mitochondrial morphology was followed by fluorescence microscopy in *bck1*∆ (RSY1050) and *bck1*∆ *cnc1*∆ (RSY1052) cultures expressing mt-dsRed and either a vector control or *RHO1^G19V^*. **(D)** Quantitation of three independent isolates exhibiting reticular or fragmented mitochondria were determined from ≥ 600 cells per culture.

A previous study found that introduction of a constitutively active Rho1, but not Bck1, could largely restore the normal H_2_O_2_-induced transcription program to cells deleted for the CWI pathway cell wall sensor Mtl1 39,44. These results suggested to these authors that Mtl1 signals through Rho1 but not downstream components of the CWI pathway. We previously demonstrated that Mtl1 and the CWI signaling are required for normal cyclin C nuclear release and subsequent mitochondrial fragmentation 27,28. Therefore, we tested whether activated Rho1 alone was sufficient to regulate cyclin C translocation in the absence of additional stress signals. To address this question, we examined mitochondrial morphology by fluorescence microscopy in cells expressing either Rho1^G19V^ or a vector control. This experiment revealed that mitochondria were significantly more fragmented in cells expressing Rho1^G19V^ than the vector control (Figure 6A, quantitated in 6B). These results indicate that Rho1 signaling is sufficient to induce mitochondrial fragmentation. To determine if this phenotype is dependent on cyclin C, this experiment was repeated in a *cnc1*∆ strain. In the absence of cyclin C, mitochondrial fragmentation was significantly reduced in cells expressing Rho1^G19V^ compared to control. However, mitochondrial fragmentation was still observed above vector controls in the *cnc1*∆ strain indicating that Rho1 signaling is able to induce mitochondrial fragmentation in both cyclin C dependent and independent mechanisms.

We have previously demonstrated that activation of the CWI MAPK pathway using activated Rho1 is translated by the cells as a high stress situation 27,28. Therefore we addressed if the Rho1^G19V^ mediated mitochondria fragmentation seen is dependent upon Bck1. The results (Figure 6C and quantitated in 6D) show that this is indeed the case with over 60% of *bck1*∆ cells harboring Rho1^G19V^ showing cyclin C dependent mitochondrial fission compared to ~10% of the cells harboring a vector control. These results support the model that, under conditions of high stress, the stress signal can reach cyclin C independent of Bck1.

### Rho1-dependent cyclin C translocation induces oxidative stress hyper-sensitivity.

**Figure 7:**
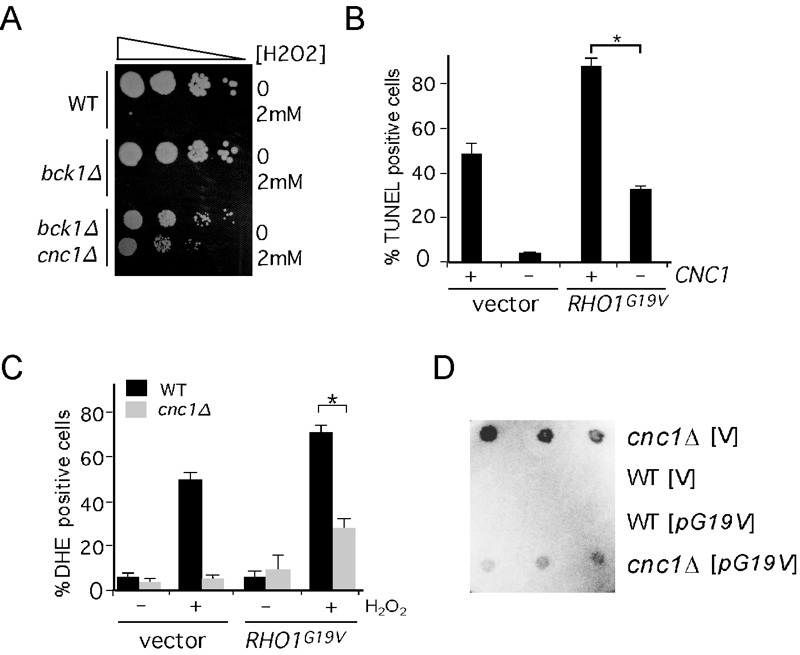
FIGURE 7: *RHO1^G19V^* promotes cyclin C-dependent programmed cell death in the absence of stress. **(A)** Cell viability assays on wild type (RSY10), *bck1*∆ (RSY1050) and *bck1*∆* cnc1*∆ (RSY1052) strains following treatment with 2 mM H_2_O_2_ for 2 h. Decreasing dilutions of the cells (represented by the arrow) were plated on YPDA media and the surviving colonies photographed after 2 days at 30°C. **(B)** Strains wild type (RSY10) and *cnc1*∆ (RSY391) cultures expressing mt-dsRed and either a vector control or *RHO1^G19V^* were treated with 2 mM H_2_O_2_ for 20 h then assayed for double stranded breaks using TUNEL assays. The percent of TUNEL positive cells is given (mean ± s.e.m.). In all panels, the asterisk indicate p < 0.05 (Student’s T test). **(C)** Strains described in (A) were treated with 2 mM H_2_O_2_ for 20 h and the percent of the population positive for DHE oxidation is shown (mean ± s.e.m.). **(D)** Cyclin C represses transcription in Rho1 hyper-activated strains. The aberrant vegetative expression of the meiosis-specific *spo13-lacZ* reporter gene (p42513Z) was analyzed in wild type (RSY10) and *cnc1*∆ (RSY391) strains harboring a vector control (V) or hyperactive *RHO1^G19V^* allele. Three independent transformants were assayed on plates for ß-galactosidase expression by cleavage of the substrate 5-bromo-4-chloro-3-indolyl-ß-D-galactopyranoside (X-gal).

Extensive mitochondrial fission is an early cytological landmark in PCD initiation, although fragmentation itself does not insure cell death 45. We have previously demonstrated that precocious release of cyclin C from the nucleus caused mitochondrial fission in the absence of stress and sensitized cells to H_2_O_2_ 46. To determine whether Rho1-dependent cyclin C translocation also induced oxidative stress hyper-sensitivity, we monitored the viability of *bck1*∆ cells following treatment with 1.2 mM H_2_O_2_. As expected from our earlier results, loss of Bck1 activity did not alter the stress sensitivity compared to wild type (Figure 7A). This loss of viability was dependent on cyclin C as the *bck1*∆* cnc1*∆ double mutant displayed resistance to H_2_O_2_. To determine whether the cell death observed on the plates were due to PCD, the strains just described were treated with H_2_O_2_ and PCD execution was measured by TUNEL assays. These experiments indicated that, similar to mitochondrial fission, activated Rho1 increased H_2_O_2_ sensitivity that was partially dependent on cyclin C (Figure 7B).

To understand the nature of this additional signal, the percentage of the population exhibiting high ROS concentrations was determined by DHE staining and fluorescence activated cell analysis. DHE oxidation measures internal ROS levels that can be elevated by damaged mitochondria and elevated DHE oxidation is casually related to the cell’s likelihood to induce PCD. The results indicated that the presence of Rho1^G19V^ did not induce elevated ROS levels under normal growth conditions but the percent of the population of DHE positive cells increased following H_2_O_2_ treatment (Figure 7C). As observed with the TUNEL assays, much, but not all, of the increase in DHE positive cells is dependent on cyclin C. The cyclin C-independent ROS increase may be the result of continued mitochondrial fragmentation through another Rho1-dependent signaling pathway (see discussion).

The partial requirement of cyclin C for Rho1^G19V^-dependent mitochondrial fission, H_2_O_2_-induced PCD and elevated internal ROS levels could be due to its transcriptional regulatory role, its mitochondrial function, or both. Given its reduced overall levels in cells expressing activated Rho1, we first asked whether cyclin C was still functioning as a transcription factor. Cyclin C represses the transcription of both stress response 33 and early meiotic genes (e.g., *SPO13*) 47 during mitotic cell division. Therefore, wild type and *cnc1*∆ strains were transformed with plasmids expressing either *RHO1^G19V^* or a vector control and a *spo13*-lacZ reporter gene. Using a plate assay to monitor ß-galactosidase activity, this experiment revealed that cyclin C still repressed *spo13*-lacZ transcription even in the presence of Rho1^G19V^ (Figure 7D). These results indicate that although cyclin C levels are reduced, it must still pass through the nucleus with sufficient retention to repress *SPO13* transcription. In addition, these findings suggest that the role cyclin C plays in enhancing the activated Rho1 phenotypes can occur through its transcriptional and/or mitochondrial role (see Discussion).

### Ste11 is required for cyclin C mitochondrial relocalization and destruction in response to high H_2_O_2_ induced stress.

**Figure 8:**
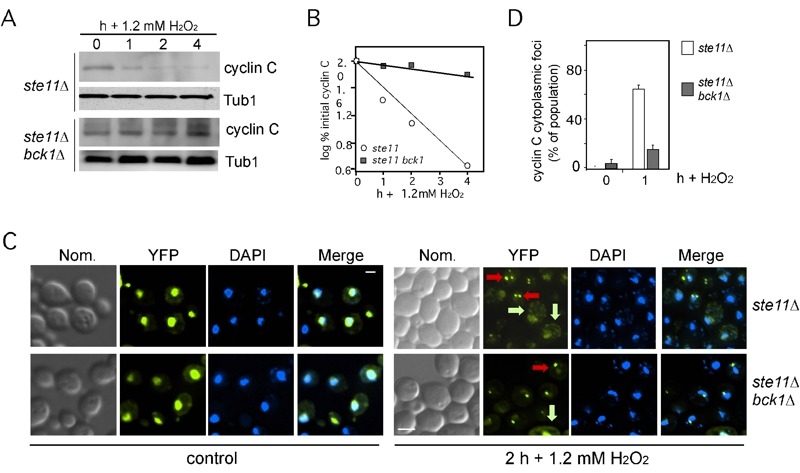
FIGURE 8: Ste11 is functionally redundant with Bck1 following 1.2 mM H_2_O_2_ stress. **(A)** Myc-cyclin C levels were monitored as described previously in *ste11*∆ (RSY1983) and *ste11*∆* bck1*∆ strain (RSY1985) exposed to 1.2 mM H_2_O_2_ for the times indicated. Tub1 levels were used as a loading control. **(B)** Quantification of the results obtained in (A). **(C)** Ste11 is required for normal cyclin C nuclear release following 1.2 mM stress. Fluorescence microscopy was used to monitor cyclin C-YFP localization in *ste11*∆ (BLY21) and *bck1*Δ* ste11*∆ (BLY478) cells before (0 h) and following (2 h) 1.2 mM H_2_O_2_ treatment. Representative images (collapsed de-convolved 0.2 µm slices) of the results obtained are shown. Perinuclear and cytoplasmic localization of cyclin C-YFP are indicated by the red and yellow arrows, respectively. **(D)** Quantification of the results in (C). The percent of the population displaying at least 3 cyclin C-YFP foci in the cytoplasm is given (mean ± s.e.m.). At least 200 cells were counted per time point from 3 individual isolates.

Previous studies revealed a genetic interaction between the CWI pathway and Ste11 function. Specifically, these studies found that a *ste11*∆* bck1*∆ double mutant displayed synthetic defects in cell wall integrity and that Slt2 is required for Ste11-dependent regulation of* FKS2* 14,15. To test whether Ste11 played a role in cyclin C destruction in response to high H_2_O_2_ treatment, a *ste11*∆ and a *bck1*∆* ste11*∆ strain was constructed expressing myc-cyclin C. A time-course experiment revealed that cyclin C was protected from destruction in the double mutant whereas it was destroyed with similar kinetics to wild type cells in *ste11*∆ (Figure 8A, quantitated in 8B). Next, cyclin C localization was monitored under high H_2_O_2_ conditions. A *ste11*∆ single mutant or the *bck1*∆* ste11*∆ double mutant was transformed with the cyclin C-YFP expression plasmid. In the absence of stress, cyclin C-YFP exhibited a diffuse nuclear signal in both strains similar to wild type (Figure 8C). In the *ste11*∆ mutant, exposure to high H_2_O_2_ concentrations resulted in cells displaying either cytoplasmic (light green arrows) or large peri-nuclear foci (red arrows). These results indicate that Ste11 is not essential for cyclin C relocalization but appears necessary for high efficiency translocation. In the double mutant, a higher percentage of the population exhibited a large focus associated with the nucleus and fewer cytoplasmic foci (Figure 8C, quantified in Figure 8D and see Figure S2 for field view of cells). These results indicate that Ste11 and Bck1 play partially redundant roles in directing cyclin C from the nucleolus to the cytoplasm in response to high H_2_O_2_ treatment.

## DISCUSSION

Cyclin C and its kinase Cdk8 are components of the RNA polymerase II associated Mediator complex. This kinase functions primarily as a transcriptional repressor and targets a variety of genes most frequently those involved in the stress response. In response to stress, this repression is removed by nuclear to cytoplasmic relocalization of cyclin C. Previous studies found that the MAPKKK component of the CWI pathway (Bck1) is essential to trigger cyclin C relocalization in response to low-level H_2_O_2_ exposure. This report extends these observations by demonstrating that, in response to either high H_2_O_2_ concentrations or in the presence of the constitutively active Rho1, Bck1 activity is combined with that of Ste11, the MAPKKK normally associated with mating type, pseudohyphal or high osmolarity environmental signaling. With respect to the cyclin C relocalization pathway, this study found that Ste11 and Bck1 are required for cyclin C translocation from the nucleolus to the cytoplasm. Taken together, these results revealed a mechanism by which the cell can distinguish low from high oxidative stress conditions by Ste11 activation. In addition, these results demonstrate a regulated step in the cyclin C relocalization pathway from the nucleolus to the cytoplasm.

MAP kinase signaling pathways are designed to recognize specific stimuli then transduce that signal to the nucleus to alter the transcription program. To achieve this goal, four general mechanisms (docking interactions, scaffold proteins, cross pathway inhibition and kinetic insulation) are in place to insulate the activation of one pathway from another (reviewed in 8). However, recent studies have revealed an increasing important role for allowing MAP kinase pathway crosstalk. For example, in response to zymolyase cell wall stress, the sensors of the HOG pathway are required to upregulate genes controlled by the CWI MAPK module (48,49 and reviewed in 50). Likewise, in strains deleted for either Pbs2 (MAPKK and scaffold) or Hog1 (see Figure 1), stimulation with high osmolarity stress results in crosstalk to the mating response 51. These observations are consistent with the hypothesis of Murray and colleagues who posited that connectivity between different MAPK signaling pathways allows for multiple input sensing and decision making 52.

**Figure 9:**
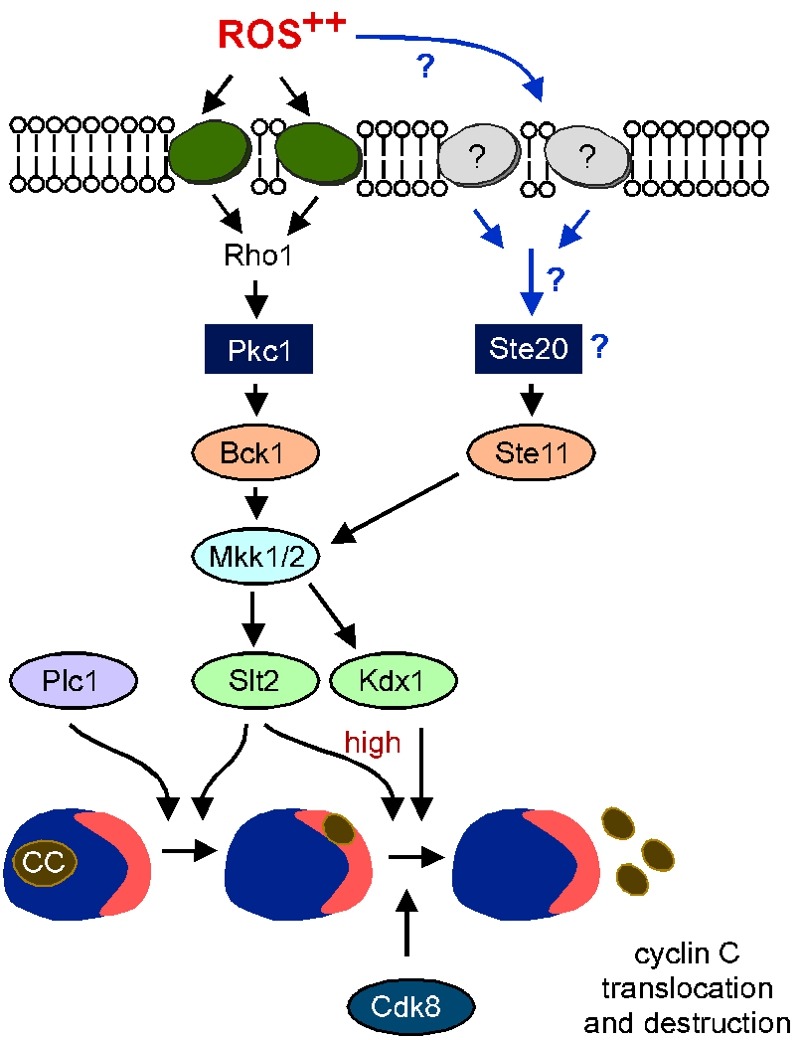
FIGURE 9: Proposed model for stress-activated signal transduction pathways mediating cyclin C nuclear export and destruction following high oxidative stress. Following high ROS stress cyclin C moves first to the nucleolus and then to the cytoplasm. Movement to the nucleolus requires Plc1 55 whereas nucleolar export requires Slt2, Kdx1 activation and Cdk8 function 28,34. Under mild ROS stress the canonical CWI pathway is sufficient to transmit the stress signal to cyclin C leading to its nuclear export and destruction 28. Under elevated ROS stress an additional MEKK (Ste11) is also required to transmit the stress signal via Mkk1/2 to cyclin C. Other sensors may also feed into the Ste11-Mkk1/2 pathway (see discussion for details).

In this report, we identified a lateral signaling mechanism for the cell wall integrity (CWI) pathway that is activated in response to excessive oxidative stress. Specifically, Ste11 is required for normal degradation and cytoplasmic relocalization of cyclin C in response to high stress conditions (see Figure 9). The genetic, cellular and biochemical results presented here suggest that mechanistically this is achieved by Ste11 activating Mkk1/2. Mkk1/2 in turn activates Slt2 and Kdx1, an event that is required for cyclin C translocation 28. Importantly, to date the redundant Mkks are the only reported MAPKKs that can transfer the activated phosphate group to Stl2 and Kdx1 (reviewed in 6). Previous studies have also reported functional redundancy between Ste11 and Bck1 with regards to the activation of *FSK2 *following stress 14. Interestingly, Mkk1/2 is not the only kinase that Ste11 activates under high oxidative stress. Tyrosine phosphorylation of Hog1 has been observed following treatment of cells with 3 mM H_2_O_2_ 53. Similarly, by using expression quantitative trait loci (eQTL) studies, Wang *et al.* proposed that Ste11 may also regulate the Pbs2-Hog1 signaling pathway under oxidative stress 54. However, it is unlikely that H_2_O_2_ induced activation of Hog1 is required for cyclin C translocation to the cytoplasm as cyclin C is degraded in response to low H_2_O_2_ stress in *hog1*∆ cells 55.

One unanswered question that has arisen from this work is how the stress signal is transmitted to Ste11 following high H_2_O_2_ stress (blue arrows, Figure 9). Our previous work has suggested that additional cell wall sensors are activated in response to high stress 27. One strong possibility is that similar to zymolyase stress, the sensors from the HOG pathway, Sln1 and/or Sho1, may transmit the oxidative stress signal to Ste11. Consistent with this, it has been reported that Ste11 can bind directly to the cytoplasmic tail of Sho1 56 although it is not clear how this interaction directs kinase activation. Also, in support of this model is the observation that in pathogenic fungi *Aspergillus fumigatus *57 and* Candida albicans *58), both Sho1 and Sln1 are cell wall sensors for environmental oxidative stress 59. Consistent with the notion that both sensor proteins play a role, deletion of *SHO1* in the *bck1*∆ strain resulted in an "intermediate" phenotype with respect to cyclin C nuclear export and degradation (Figure S3). In other words, cyclin C was partially stabilized in the *bck1*∆* sho1*∆ strain and was exported but not as robustly as in the *sho1*∆ mutant alone. However, the double mutant did not protect cells from dying following treatment with H_2_O_2_.

The nucleolus is the site of rRNA transcription and ribosome assembly. In response to many stressors including oxidative stress, rRNA transcription and ribosome assembly is halted. For example, the RNA polymerase I transcription factor Rrn3, relocalizes from the nucleolus to the cytoplasm to inhibit transcription following cellular damage 60. In mammalian cells, the Rrn3 homolog TIF-1A is also inhibited in response to stress through phosphorylation by the stress activated Jnk MAPK 61. This report demonstrated that cyclin C transits through the nucleolus in response to oxidative stress prior to its translocation to the cytoplasm. We have previously found that Cdk8 also exhibits a change from diffuse nuclear to the nucleolus following H_2_O_2_ treatment 34. These results raise the possibility that cyclin C-Cdk8 performs a regulatory role in the stressed nucleolus. No target of cyclin C-Cdk8 in the RNA Pol I machinery has been identified but this kinase has been shown to modify components of the RNA Pol II Mediator complex, transcription factors and chromatin binding proteins 62. Therefore, a role for cyclin C-Cdk8 in down regulating Pol I would not be surprising.

This study and previous reports 27,28,34,35,46 have begun to define the pathway that governs cyclin C translocation. Under normal conditions, cyclin C resides in the nucleoplasm as expected for a transcription factor (Figure 9). In response to stress, cyclin C relocalizes to the nucleolus through a process that requires Phospholipase C (Plc1) and a cis-acting element (A110) on the cyclin itself 34. In addition, under low stress conditions, Slt2 is also required for this step and does so by directly phosphorylating cyclin C 28. Increasing H_2_O_2_ concentrations or the presence of activated Rho1 relieves this requirement. Once cyclin C reaches the nucleolus, the combined activity of Cdk8 35, Bck1 and Ste11 (this report) are necessary for efficient translocation to the cytoplasm where it interacts with the mitochondria. Taken together, these studies define a complicated, multi-step switch that controls subcellular cyclin C localization. However, it is important to note the consequences to the cell if cyclin C is mis-localized. For example, deleting *CNC1* results in no cytoplasmic cyclin C and significant protection from oxidative stress-induced PCD 26. Conversely, loss of Med13, the nuclear anchor for cyclin C, allows continuous nuclear release of cyclin C resulting in constitutive mitochondrial fragmentation and hyper-sensitivity to oxidative stress 46. Therefore, the cell must possess a responsive, interactive system to precisely control cyclin C subcellular localization.

## MATERIALS AND METHODS

### Yeast strains and plasmids

**Table 1:** Yeast strains used in this study. ^ a^genotype: *MAT***a** *ade2 ade6 can1-100 his3-11,15 leu2-3,112 trp1-1 ura3-1* ^b^genotype: *MAT***α** *ade2-1 ade3-1 can1-100 his3-11,15 leu2-3,112 trp1-1 ura3-1* ^c^genotype homozygous diploid *ura3-52 trp1∆1 his3*

**Strain**	**Genotype**	**Source**
RSY10^a^	*MAT***a ***ade2 ade6 can1-100 his3-11,15 leu2-3,112 trp1-1 ura3-1*	47
RSY391^a^	*cnc1∆::LEU2*	33
RSY1006^a^	*slt2∆::his5+*	26
RSY1050^a^	*bck1∆::his5^+^*	This study
RSY1052^a^	*bck1∆::his5^+ ^cnc1::LEU2*	This study
RSY1736^a^	*kdx1∆::KANMX6*	28
RSY1737^a^	*kdx1∆::KANMX6 slt2::his5+*	28
RSY1988^a^	*sho1∆::KANMX4*	This study
RSY1989^b^	*sho1∆::KANMX4 bck1:: his5^+^*	This study
BLY21^b^	*ste11∆*	14
BLY478^b^	*ste11∆ bck1::URA3*	14
FLY1589^ c^	*NOP1-CFP::KANMX6*	Frank Luca personal communication

The haploid strains used in this study are listed in Table 1 and derived from RSY10, an *ade6* derivative of W303-1A 47. BLY21 (*ste11*∆*)* and BLY478 (*ste11*∆* bck1*∆) are isogenic W3031-A strains except that *ade3-1* is mutated instead of *ade6*. FLY1589 was a gift from F. Luca and is a diploid strain with integrated Nop1-CFP::*KANMX6*. *BCK1* was deleted from yeast strains RSY10 and RSY391 by using homologous recombination 63 to create RSY1050 (*bck1*∆) and RSY1052 (*bck1*∆* cnc1*∆) respectively. Strains RSY1988 (*sho1*∆) and RSY1989 (*bck1*∆* sho1*∆) were constructed by integrating the PCR amplified KANMX4 deleted *sho1* allele, obtained from the Research Genetics deletion strain collection, into RSY10 and RSY1050 respectively. In accordance with the Mediator nomenclature unification effort 64, we will use cyclin C (*SSN8/UME3/SRB11*) and Cdk8 (*SSN3/UME5/SRB10*) gene designations, respectively. Details about the plasmids used in this study can be found in Table 2. Plasmids pKC337 (*ADH1*-myc-cyclin C), and pBK37 (*ADH1*-cyclin C-YFP) are functional and have been previously described 27,33,34. The initial *BCK1-20 *construct (that was used to create pLR106) was a gift from D. Levin. The other constitutively activated alleles (*RHO1^G19V^* and *PKC1^R398P^*) plasmids were gifts from Y. Ohya and M. Hall, respectively. The plasmid used to visualize mitochondria (mt-DsRed) was a gift from J. Shaw. The Nop1-RFP plasmid was a gift from M. Lisby. The *spo13*-lacZ fusion plasmid that exhibits meiosis-specific regulation similar to that of *SPO13 *65 was a gift from R. Strich.

**Table 2:** Plasmids used in this study.

**Plasmid Name**	**Gene**	**Epitope Tag**	**Marker**	**Promotor**	**2µ/CEN**	**Reference**
pKC337	*CNC1*	1 myc	*TRP1*	*ADH1*	CEN	33
pBK37	*CNC1*	YFP	*TRP1*	*ADH1*	CEN	27
pLR106	*BCK1-20*	No	*HIS3*	*ADH1*	CEN	26
pYO964	*RHO1^G19V^*	No	*URA3*	*RHO1*	CEN	39
PKC1*	*PKC1^R398P^*		*URA3*	*PKC1*	2µ	17
Mt-DsRed	Mito-targeting	dsRed	*TRP1*	*ADH1*	2µ	68
Spo13-HIS3	*spo13*-LacZ		*HIS3*	own	CEN	65
Nop1-RFP	*Nop1*	RFP	*URA3*	own	2µ	69

### Cell growth and culturing conditions

Cells were grown in either rich, non-selective medium (YPDA) or synthetic minimal medium (SC) to allow for plasmid selection as previously described 33. The *spo13-*lacZ plate assays were conducted as previously described 47,66. In short, yeast cells were grown on filter paper (3MM, Midwest Scientific) placed on agar medium selective for the plasmids. Filters were then lifted and frozen in liquid nitrogen to rupture the cells then overlayed with agar containing 5-bromo-4-chloro-3-indolyl-β-D-galactopyranoside (X-Gal). After 2 to 36 h, color development was stopped by air drying.

### Survival and stress assays

For all H_2_O_2_ stress assays, cells were grown to mid-log phase (6 x 10^6^ cells/ml) in minimal media and then treated with H_2_O_2_ at the concentrations and times written. Cell survival, TUNEL and DHE assays were conducted exactly as previously described 35,46. For both DHE and TUNEL assays, positive cells were detected using flow cytometry analysis (30,000 cells counted per time point). All statistical analysis was performed using the Student’s t test with p < 0.05 considered significant. All analyses were conducted with at least three independent cultures with 200 or more cells counted per time point.

### Immunofluorescence microscopy and mitochondrial fission/fusion assays 

YFP-cyclin C subcellular localization was monitored and scored as described previously 28. For all experiments, the cells were grown to mid-log (5 x 10^6^ cells/ml), treated with the concentration of H_2_O_2_ and for the time points indicated. The cells were harvested, fixed in 4% para-formaldehyde for 30 min, washed three times in water, stained with DAPI and then analyzed by fluorescence microscopy. Images were obtained using a Nikon microscope (model E800) with a 100 X objective (Plan Fluor Oil, NA 1.3) and a CCD camera (Hamamatsu model C4742). Data were collected using Autoquant^®^ and processed using Image Pro software. All images of individual cells were optically sectioned (0.2 µm slices at 0.3 µm spacing) and deconvolved and the slices were collapsed to visualize the entire fluorescent signal within the cell. Cyclin C-YFP foci were scored as being cytoplasmic when 3 or more foci were observed outside of the nucleus. Mitochondrial fission assays were performed on live cells as described 35. In brief, mitochondrial fission was scored positive if no reticular mitochondria were observed that transversed half the cell diameter. Fusion was scored when cells exhibited one or more reticular mitochondria the diameter of the cell. Fission and fusion was scored for 200 cells from three independent isolates. Statistical analysis was performed using the Student’s T-test with p < 0.05 used to indicate significant differences. Live single cell imaging (Figure 4A) was accomplished by resuspending cells expressing cyclin C-YFP and Nop1-CFP in 1% low melting point agarose, 1 X complete minimal medium, 100 nM MitoTracker Red CMXRos (Molecular Probes) and 0.8 mM H_2_O_2_. Images were obtained immediately and every minute starting at 30 min. In all panels, the bar = 5 µm unless otherwise stated.

### Western blot analysis

Extracts prepared for analyzing myc-cyclin C levels were prepared from mid-log cultures (6 x 10^6^ cells/ml) as described previously 33 except that the lysis buffer used was 150 mM NaCl, 50 mM Tris-HCl pH 8.0, 1% NP-40, 0.15% deoxycholic acid sodium salt, 1 µg/ml pepstatin, 1 µg/ml leupeptin, 0.2% Protease Inhibitor cocktail (Sigma). In brief, 500 µg of soluble extract was immunoprecipitated using either α-myc or α-HA antibodies (Roche), collected on agarose A beads and analyzed by Western blot. Western blot signals were detected using goat α-mouse secondary antibodies conjugated to alkaline phosphatase (Sigma) and the CDP-Star chemiluminescence kit (Tropix). Signals were quantitated by phosphorimaging (Kodak Inc.). Half-life determinations were calculated by linear regression analysis with curves possessing r values > 0.9. Cyclin C levels were standardized to Tub1 levels before comparing to other values. For Slt2 phosphorylation assays, protein extracts were made using the LiOAc/NaOH extraction procedure exactly as described 67. Slt2 phosphorylation was detected using α-phospho-p44/42 antibodies (Cell Signaling) as previously described 28. Slt2 was detected using α-Mpk1 antibody (sc20168, Santa Cruz). Tub1 was visualized alpha-tubulin antibodies (12G10) were obtained from the Developmental Studies Hybridoma Bank (Univ. of Iowa).

## SUPPLEMENTAL MATERIAL

Click here for supplemental data file.

All supplemental data for this article are also available online at http://microbialcell.com/researcharticles/the-mapkkks-ste11-and-bck1-jointly-transduce-the-high-oxidative-stress-signal-through-the-cell-wall-integrity-map-kinase-pathway/.
