# Supplementary material for: The MAPKKKs Ste11 and Bck1 jointly transduce the high oxidative stress signal through the cell wall integrity MAP kinase pathway
Source: Microb Cell. 2015 Sep 7;2(9):329–42. doi: 10.15698/mic2015.09.226 (PMC4850913; doi:10.15698/mic2015.09.226)
Supplement: Supplementary file 1 [file mic-02-329-s01.pdf]

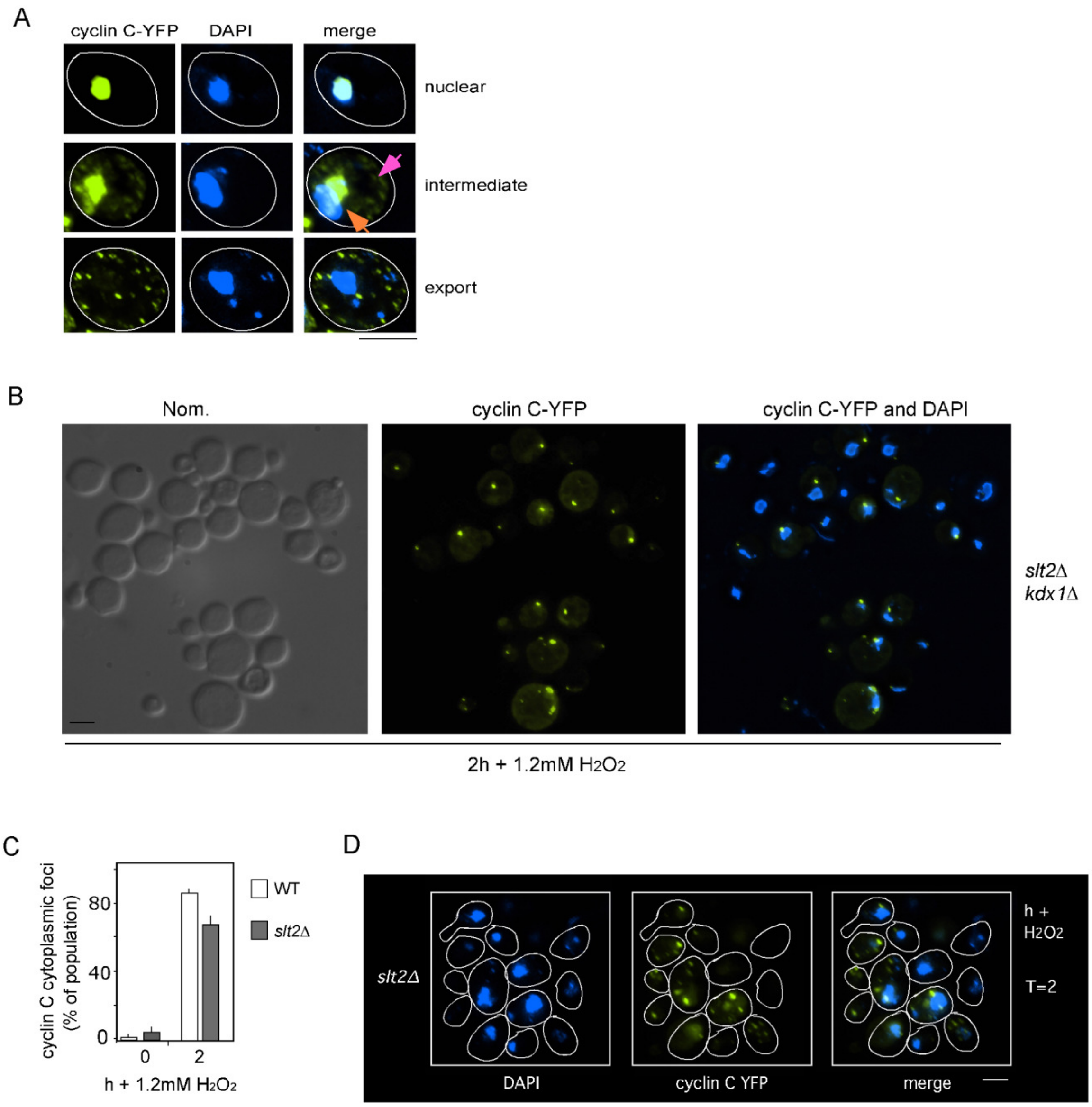

FIGURE S1: Cyclin C is exported from the nucleus in *slt2Δ* cells under high stress. (A) Representative images of the progression of cyclin C-YFP export in DAPI stained wild type cells. In the absence of stress cyclin is located in the nucleus (upper panels). The “intermediate” phenotype (middle panels) is characterized by cyclin C being predominantly located just alongside the nucleus (orange arrow) cyclin C and well being discreet foci in the cytoplasm (pink arrow). The “export” phenotype (bottom panels) is characterized by cyclin C being predominantly exported from the nucleus and located in the cytoplasm. **(B)** Field view (collapsed deconvolved 0.2 µM slices) of fluorescence microscopy conducted on mid-log phase on *slt2Δ kdx1Δ* (RSY1737) cells expressing YFP-cyclin C (pBK37) following (2 h) 1.2 mM H_2O_2 treatment. The cells were fixed, stained with DAPI and then examined by fluorescence microscopy. **(C)** The percent of wild type (RSY10) and *slt2Δ* (RSY1006) cells (mean ± s.e.m.) within the population displaying at least 3 cytoplasmic cyclin C foci is given before and following H_2O_2 (0.4 and 1.2 mM) treatment for 2 h. At least 200 cells were counted per timepoint from 3 individual isolates. **(D)** Representative images (collapsed de-convolved 2µ slices) of *slt2Δ* cells harboring cyclin C-YFP after 2 h 1.2mM H_2O_2 stress.

A

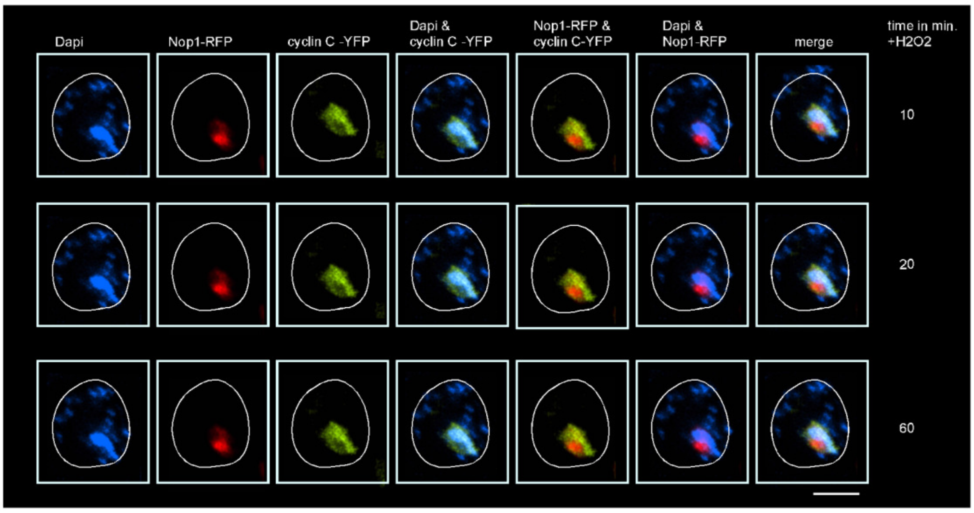

B

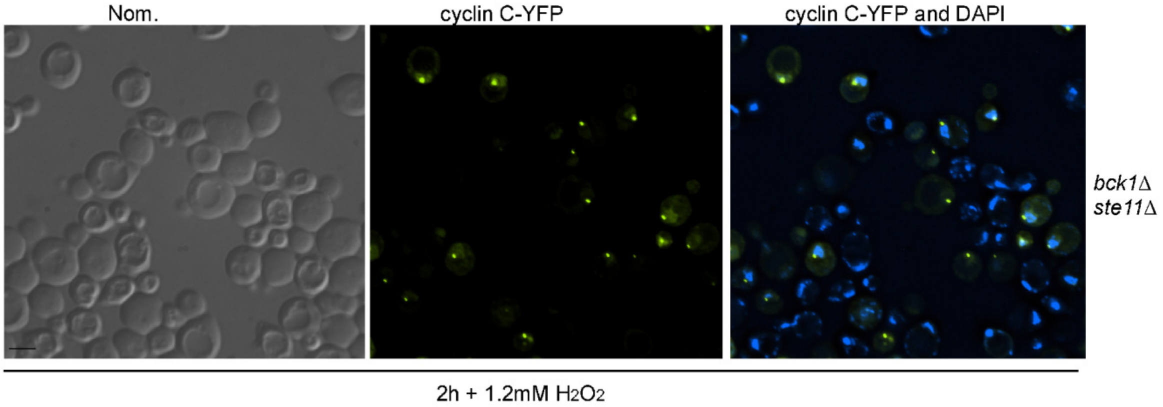

FIGURE S2: (A) Fixed cell images of the whole cell wild type cells (RSY10) expressing cyclin C-YFP and Nop1-RFP. Cells were harvested and stained with DAPI following 1.2mM H_2O_2 at the time points indicated. **(B)** Field view (collapsed deconvolved 0.2 µM slices) of fluorescence microscopy conducted on mid-log phase on *bck1Δ ste11Δ* (BLY478) cells expressing YFP-cyclin C (pBK37) following (2 h) 1.2 mM H_2O_2 treatment. The cells were fixed, stained with DAPI and then examined by fluorescence microscopy.

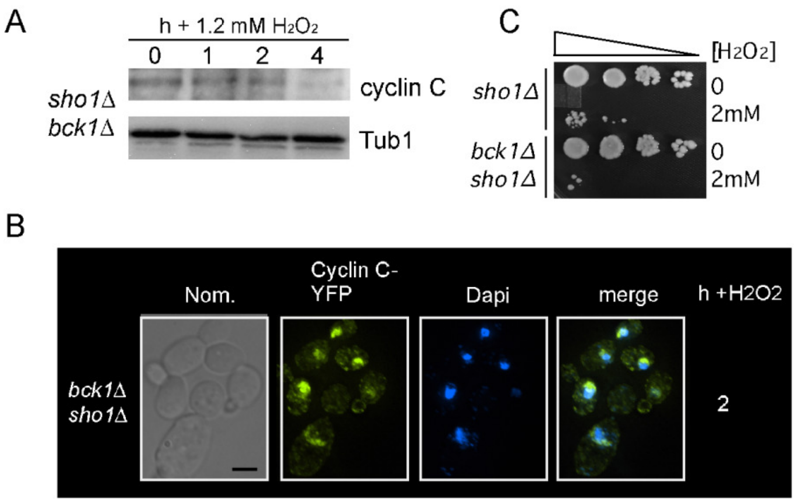

FIGURE S3: the cell wall sensor Sho1 may be able to transmit the stress signal to Ste11 following H_2O_2 stress. **(A)** *sho1Δ* (RSY1988) and *bck1Δ sho1Δ* (RSY1989) cultures expressing myc-cyclin C (pLR337) were grown to mid-log phase (0 hr) then treated with 1.2 mM H_2O_2 for the indicated times. Cyclin C levels were determined by Western blot analysis of immunoprecipitates. Tub1 levels were used as a loading control. **(B)** Cell viability assays on wild type *sho1Δ* and *bck1Δ sho1Δ* strains following treatment with 2mM H_2O_2 for 2 hrs. Decreasing dilutions of the cells (represented by the arrow) were plated on YPDA media and the surviving colonies photographed after 2 days at 30°C. **(C)** Cyclin C exhibits the "intermediate" localization phenotype in the *bck1Δ sho1Δ* strain following 1.2 mM H_2O_2 stress. Fluorescence microscopy was conducted on mid-log phase on *bck1Δ sho1Δ* cells expressing YFP-cyclin C (pBK37) following (2 h) 1.2 mM H_2O_2 treatment. The cells were fixed, stained with DAPI and then examined by fluorescence microscopy. Representative images (collapsed deconvolved 0.2 µM slices) of the results obtained are shown.
